# Assessing scalability of an intervention: why, how and who?

**DOI:** 10.1093/heapol/czz068

**Published:** 2019-07-31

**Authors:** Karen Zamboni, Joanna Schellenberg, Claudia Hanson, Ana Pilar Betran, Alexandre Dumont

**Affiliations:** 1 Department of Disease Control, Faculty of Infectious Tropical Diseases, London School of Hygiene and Tropical Medicine, Keppel Street, London, UK; 2 Department of Public Health Sciences, Karolinska Institutet, Nobels väg 6, Solna and Alfred Nobels Allé 8, Huddinge, Stockholm, Sweden; 3 Department of Reproductive Health and Research, World Health Organization, 20 Avenue Appia, 2011 Geneva, Switzerland; 4 CEPED, IRD, Université de Paris, Equipe SAGESUD, ERL INSERM U 1244, 45 Rue des Saints Pères, Paris, France

**Keywords:** Scale-up, scalability, evaluation

## Abstract

Public health interventions should be designed with scale in mind, and researchers and implementers must plan for scale-up at an early stage. Yet, there is limited awareness among researchers of the critical value of considering scalability and relatively limited empirical evidence on assessing scalability, despite emerging methodological guidance. We aimed to integrate scalability considerations in the design of a study to evaluate a multi-component intervention to reduce unnecessary caesarean sections in low- and middle-income countries. First, we reviewed and synthesized existing scale up frameworks to identify relevant dimensions and available scalability assessment tools. Based on these, we defined our scalability assessment process and adapted existing tools for our study. Here, we document our experience and the methodological challenges we encountered in integrating a scalability assessment in our study protocol. These include: achieving consensus on the purpose of a scalability assessment; and identifying the optimal timing of such an assessment, moving away from the concept of a one-off assessment at the start of a project. We also encountered tensions between the need to establish the proof of principle, and the need to design an innovation that would be fit-for-scale. Particularly for complex interventions, scaling up may warrant rigorous research to determine an efficient and effective scaling-up strategy. We call for researchers to better incorporate scalability considerations in pragmatic trials through greater integration of impact and process evaluation, more stringent definition and measurement of scale-up objectives and outcome evaluation plans that allow for comparison of effects at different stages of scale-up.

**Key Messages**

- We developed a scalability assessment during the design of a multi-component intervention to reduce unnecessary caesarean sections in low- and middle-income countries, adapting available scale-up frameworks and tools.
- We documented the methodological challenges we encountered. These include: achieving consensus on the purpose of a scalability assessment; identifying the optimal timing of such an assessment; and resolving tensions between the need to establish the proof of principle, and the need to design an innovation that would be fit-for-scale.
- As scale-up is a relatively new focus for implementation research, we found little evidence that these methodological challenges have been fully addressed. We call for researchers to better incorporate scalability considerations in pragmatic trials through greater integration of impact and process evaluation, more stringent definition and measurement of scale-up objectives and outcome evaluation plans that allow for comparison of effects at different stages of scale-up.

## Introduction

Planning for scale is increasingly important to increase impact and achieve health goals (Implementing Best Practices Consortium, 2007), and there is growing recognition that publications, policy reform and training alone are insufficient to achieve scale (ExpandNet WHO, 2009; Edwards, 2010; Barker *et al.*, 2016; Wright *et al.*, 2018). For complex interventions, understanding conditions that may facilitate their implementation at scale is increasingly important.

Concurrently with the growing focus on scale-up in global health, the body of literature on scale-up has expanded in the last decade. Previous research helped distinguish the concept of scale-up from replication and expansion, and made theoretical assumptions around scale-up explicit, borrowing largely from Roger’s diffusion of innovation theory and Glaser’s formulation of factors related to knowledge transfer (Glaser *et al.*, 1983; Mangham and Hanson, 2010; Fixsen, 2013; Rogers, 2013). More recently, empirical research has focused on the process of scale-up, and on identifying factors facilitating or hindering it, with evidence emerging from diverse fields, including reproductive health, malaria and HIV/AIDS, and diverse settings, including both low–middle income (Wall *et al.*, 2009; Bradley *et al.*, 2012; Spicer *et al.*, 2014; Dickson *et al.*, 2015; Smith *et al.*, 2015; Perez-Escamilla and Moran, 2016) and high-income countries (McCannon and Perla, 2008; Milat *et al.*, 2015; Aldbury *et al.*, 2018; January 2018). Generic models and frameworks to plan scale-up efforts during intervention delivery are available in the literature, often accompanied by case studies of projects or initiatives that reached scale (ExpandNet WHO, 2009, 2010; Yamey, 2011; Cooley and Kohl, 2012; Barker *et al.*, 2016; Milat *et al.*, 2016). These have mostly emerged from experiences in low- and middle-income countries, with one exception (Milat *et al.*, 2016).

We define scale-up in line with the WHO ExpandNet definition, as ‘deliberate efforts to increase the impact of successfully tested health innovations, so as to benefit more people and to foster policy and programme development on a lasting basis’ (ExpandNet WHO, 2009). This definition assumes that scale-up can be an intentionally guided process, as opposed to spontaneous diffusion, and emphasizes institutionalization and sustainability of innovations into a health system, as opposed to just expansion of coverage.

The literature on scale-up has also referred to failures (Glassman, 2016; Jordan *et al.*, 2016)—although negative experiences are not as widely documented—and attributed these, at least in part, to untimely consideration of the scale-up process and priorities: in other words, scale-up has often been an afterthought (Cooley and Kohl, 2006; ExpandNet WHO, 2011). Implementers are now encouraged to ‘design for scale’ or to consider intervention ‘scalability’ during pilot phases.

We defined ‘scalability’ as ‘the ability of a health intervention shown to be efficacious on a small scale or under controlled conditions to be expanded under real-world conditions to reach a greater proportion of the eligible population, while retaining effectiveness’, in line with Milat (Milat *et al.*, 2013). This definition, emerging from the health promotion field, encompasses three themes: (1) expansion of coverage, the potential reach of an intervention varying in relation to the problem being addressed, characteristics of the intervention, the target group, and the context; (2) transferring control for delivery from initial implementers or innovators to local actors or institutions; and (3) retaining the effectiveness demonstrated in proof of principle studies (Milat *et al.*, 2013). These themes differentiate the concept of ‘scalability’ from the related concepts of transferability, replicability and sustainability (Supplementary Annex S1) (Bonell, 2006).

The concept of scalability is still relatively new, and in practice it is often confused with ability to widen the reach of an intervention, without much attention to continued robust performance under routine conditions, or to the extent to which it is embedded in a local delivery system.

**Figure 1:**
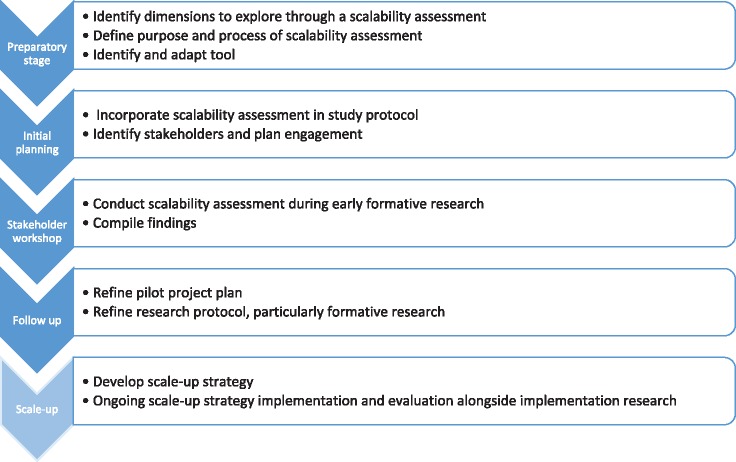
Scalability assessment process in QUALI-DEC.

This article discusses methodological lessons learned in incorporating scalability considerations during the design of a proof of principle trial to evaluate a multifaceted intervention to reduce unnecessary caesarean section rates in low- and middle-income countries (QUALI-DEC^1^, see Supplementary Box). We agreed that incorporating a scalability assessment into the QUALI-DEC protocol would help tailor the intervention and implementation approach and may increase the likelihood of success at scale. Our scalability assessment process is outlined in Figure 1. Here, we describe our experience in the preparatory and initial planning stages. We anticipate that further learning will occur as we conduct the assessment and begin implementation. We believe that such reflection is valuable to other researchers, given the limited application of the concept of scalability in research and the relative scarcity of bibliography in this area.

## Methods

First, we conducted a review and synthesis of scale-up frameworks, to identify the dimensions to explore through a scalability assessment and available tools. Based on this, we agreed on the assessment purpose and process for QUALI-DEC (Figure 1). Finally, we identified relevant tools, selected the most appropriate for our purpose and adapted it for our study.

### Review of scale-up frameworks and tools

Through a literature search in PubMed, Google (for grey literature) and references of previous reviews on similar topics, we identified 10 models or scale-up frameworks presented as a generic tool to aid scale-up beyond a specific health intervention (Table 1), of which 5 were based on implementers’ experiences, and 5 originated from the research community, mostly as literature reviews supported by qualitative interviews with stakeholders in a given health system or a Delphi process. Most were framed against Rogers’ diffusion of innovation theory (Rogers, 2013), although this was only explicitly referred to in four frameworks.

**Table 1:** Scale-up frameworks

Framework	Theoretical framing	Basis of framework	Practical application		
			Scale-up strategy tools	Scalability assessment	Purpose of scalability assessment
Massoud (2004 **)**	Not explicit	Practice	No (QI methods)	No	
Implementing Best Practices Consortium (2007 **)**	Explicit (diffusion of innovation theory)	Practice, supported by literature	No	No	
ExpandNet/WHO (2007–2012) (Simmons *et al.*, 2007; McCannon and Perla, 2008**;**ExpandNet WHO, 2009, 2010**)**	Explicit (diffusion of innovation theory and Glaser’s CORRECT attributes)	Practice, supported by literature	Yes	Yes	Ensure relevance of innovation and tailor to setting; generate political commitment; reach consensus on expectations for scale-up.
Yamey (2011 **)**	Explicit (diffusion of innovation and social network theory)	Literature review and interviews	No	No	
**Cooley/Management Systems International (** Cooley and Kohl, 2006, 2012**)**	Not explicit, but present (diffusion of innovation theory and Glaser’s CORRECT attributes)	Practice, supported by literature	Yes	Yes	Anticipate likely challenges to maximize feasibility of scale-up through adaptation.
Bradley *et al.* (2012 **)**	Not explicit, but present (diffusion of innovation theory; social cognitive theory and social networks)	Literature review and interviews	No	No	
Cambon *et al.* (2013 **)**	Not explicit	Practice	Yes	Yes	Concerned primarily with transferability/replicability.
Spicer *et al.* (2014 **)**	Not explicit	Interviews	No	No	
Barker *et al.* (2016 **)**	Explicit (diffusion of innovation theory)	Literature review, supported by practice	No (QI methods)	No	
Milat *et al.* (2016 **)**	Not explicit	Literature review,	Yes	Yes	Determine whether intervention can realistically be scaled up. Emphasizes evidence of effectiveness as precondition for scale-up.

QI = quality improvement.

We analysed frameworks to identify critical factors that require consideration when planning scale-up, and found five common themes: (1) attributes of the innovation; (2) attributes of the implementers (actors introducing an innovation or actively supporting their scale-up); (3) attributes of the adopting community; (4) socio-political context and (5) scale-up strategy (Table 2).

**Table 2:** Factors considered in scale-up frameworks

Features	ExpandNet (Simmons *et al.*, 2007; McCannon and Perla, 2008; ExpandNet WHO, 2009, 2010**)**	Management Systems International (Cooley and Kohl, 2006, 2012**)**	Yamey (2011)	Implementing Best Practices Consortium (2007)	Massoud (2004)	Barker *et al.* (2016)	Bradley *et al.* (2012)	Cambon *et al.* (2013)	Spicer *et al.* (2014), Massoud (2004**)**	Milat *et al.* (2016)
Attributes of the innovation/intervention	✓	✓	✓	✓		✓	✓	✓	✓	✓
Credibility of model (evidence base for innovation)	✓	✓	✓	✓			✓		✓	✓
Observability of results (impact or effectiveness)	✓	✓		✓			✓		✓	✓
Relevance to concern of potential adopters	✓	✓		✓		✓	✓	✓	✓	✓
Relative advantage over existing practice	✓	✓		✓		✓	✓		✓	
Simplicity or ease of adoption	✓	✓	✓	✓		✓	✓		✓	
Model testable and adaptable	✓	✓	✓	✓				✓	✓	✓
Affordability or cost-effectiveness	✓	✓						✓	✓	
Acceptability	✓						✓	✓	✓	✓
Aligned and harmonized with existing government health system or programme	✓	✓							✓	✓
Attributes of implementers	✓	✓	✓	✓	✓	✓	✓	✓	✓	✓
Leadership and credibility	✓		✓	✓	✓	✓	✓		✓	
Use of champions	✓			✓	✓		✓		✓	✓
Networking, collaboration and partnership (to foster buy-in)	✓	✓	✓	✓			✓	✓	✓	✓
Capacity to support scale-up (skills, size, resources and experience)	✓								✓	✓
Stability or grant size and length	✓								✓	
Culture of urgency and persistence				✓		✓				
Provision of capacity building for adopting stakeholders	✓								✓	✓
Attributes of adopting community	✓	✓	✓	✓	✓	✓	✓	✓	✓	✓
Clarity on who user organizations are, their needs and concerns	✓	✓					✓	✓		✓
Capacity for scale-up (staffing, skills, logistic system and other)	✓	✓			✓	✓		✓		✓
Supportive organizational culture and leadership		✓		✓						
Capacities for data collection and reporting systems		✓				✓				
Timing or window of opportunity	✓									
Learning systems	✓					✓		✓		
Engaged, activated community and institutional buy-in	✓	✓	✓					✓	✓	✓
Extent to which decision-making is data-driven									✓	
Socio-political context	✓	✓	✓		✓	✓	✓	✓	✓	✓
Political will		✓	✓				✓	✓		
Country ownership and institutional support	✓		✓		✓			✓		✓
Stakeholder analysis	✓						✓		✓	✓
Assessment of policy priorities, government systems and political climate	✓	✓							✓	
Analysis of inter-sectoral collaboration (if relevant)	✓									
Policy-legal environment (financial, economic or procedural incentives)	✓	✓			✓	✓	✓	✓		
Attitudes, values, priorities and motivations of health workers and communities	✓							✓	✓	
Scale-up strategy	✓	✓	✓	✓	✓	✓	✓		✓	✓
Create a vision for scale-up	✓	✓			✓	✓				✓
Define scalable unit					✓	✓				
Tailoring scale-up to context	✓	✓	✓	✓	✓	✓				✓
Strategic choices inform scale-up plan	✓			✓						✓
Phased approaches to scale-up or ongoing refinement for sustainability	✓	✓	✓	✓	✓	✓				
Alignment or integration in system or service	✓		✓	✓					✓	
Advocacy and communication	✓	✓		✓		✓	✓		✓	✓
Resource mobilization and alignment	✓	✓		✓						
Scale-up plan	✓			✓						✓
Ongoing M&E and dissemination of learning	✓	✓		✓	✓	✓			✓	✓

The different emphasis in focus between frameworks appeared to stem from the context and stakeholders contributing to their development. For example, the academic work was more focused on explaining how scale-up occurs and what facilitates it, while frameworks emerging from implementation were presented as practical guides to drive the process of scale-up, with a more marked focus on strategic planning. As our purpose was to identify relevant dimensions for scalability assessment, rather than to conduct a systematic review, we concluded the search once thematic saturation was achieved.

Four of the frameworks were accompanied by a tool or checklist to assess scalability during an early phase of intervention design or implementation; however, one of these (Cambon *et al.*, 2013) focused on transferability as opposed to scale-up.

### Designing a scalability assessment process

We intended to conduct an initial assessment during the pilot phase of the research, with the aims to (1) refine the intervention design to enhance scalability and (2) inform a future scale-up strategy, including advocacy and ongoing communication with key stakeholders.

The assessment was designed as qualitative and participatory, involving researchers developing and evaluating the multifaceted intervention to reduce unnecessary caesarean sections; clinicians and hospital managers in participating hospitals and Ministry of Health representatives. A stakeholder consultation workshop was proposed to be the main avenue for the assessment, after identifying a relevant scalability assessment tool.

### Tool selection and adaptation

Of the scalability tools identified in the literature, we selected Cooley and Kohl’s (2012) for our study: it was consistent with our scalability definition and developed with a LMIC setting in mind, therefore preferred to Cambon *et al.*’s, (2013) and Milat *et al.*’s, (2016) tools. Like the ExpandNet tool (ExpandNet WHO, 2011), it covered all conceptual dimensions identified in our review, and we preferred it because of its structure guiding systematic analysis of each dimension, and the specificity of its items enabling analytical depth.

We made three key adaptations to the tool: (1) we structured it in four sections, corresponding to the critical factors that require consideration to aid scale-up emerging from the evidence review: attributes of the innovation; attributes of the implementers; attributes of the potential adopting organizations or communities; and socio-political context. The fifth broad theme emerging from the review (scale-up strategy) was not included, because the findings from the scalability assessment would have been used precisely to develop a tailored scale-up strategy. (2) We omitted items that were not relevant to our intervention, for example items related to technological innovation. (3) We integrated it with dimensions from other tools: for example, from Cambon *et al.* (2013), we added items related to understanding users’ needs, to allow stronger segmentation of the project target group and a deeper understanding of the incentives and barriers to their behaviour change; and from ExpandNet WHO (2011), we added items related to attributes of the adopting organizations and community and socio-political context, for example the extent to which service delivery points in which the intervention is tested are different from those in which it would be implemented at scale.

The assessment tool was developed as a checklist, with 34 items, to be scored on a three-point scale (scale-up is easier, neutral, harder) based on participants’ perceptions and knowledge. Rather than providing a yes or no answer on whether scale-up would be possible, the assessment tool and process was designed to aid reflection on challenges and opportunities for scale-up and identify areas to be further researched or developed in later phase of the programme.

### Lessons learned

Incorporating a scalability assessment in the QUALI-DEC trial protocol raised methodological and practical challenges for the research team.

Firstly, a scalability assessment can serve both a formative purpose, i.e. to refine an intervention, and a predictive purpose, i.e. to determine the extent to which scale-up is possible. These two purposes can coexist, as donors, implementers and stakeholders in the adopting community may have an interest to identify interventions with low scalability potential early on, as this can save resources and funds. From a research perspective, achieving consensus on the purpose of a scalability assessment is necessary to improve methodological rigour. For example, emphasizing the predictive function of the scalability assessment requires further research for tool development and validation, while emphasizing the formative nature of the assessment calls for rigorous standards in participatory qualitative research to minimize bias, manage power dynamics and aid open dialogue on scalability challenges. In QUALI-DEC we defined the purpose as formative rather than predictive, interpreting scalability as an effort to maximize the intervention’s contextual fit.

Secondly, there is a need to reflect on the optimal timing. Scale-up considerations are necessary at all stages of project management, but a scalability assessment should, by definition, be integrated into early stages of intervention design and planning. In the context of QUALI-DEC, although the multiple components of the intervention were proven effective in other contexts, the lack of evidence of their effectiveness as a package in a low- or middle-income setting (which the research is designed to generate) may have led to limited the engagement from decision-makers in an early assessment. However, we also noted that greater exposure to the intervention, including understanding its components, the credibility of the evidence underpinning them, and the urgency of the problem being addressed, may have changed perceptions of its scalability over time. From a methodological point of view, a scalability assessment adds value not only early into implementation but throughout implementation, to enable ongoing analysis of scale-up barriers and opportunities. This is consistent with methodological guidance on scale-up (Cooley and Kohl, 2006; ExpandNet WHO, 2009; ExpandNet WHO, 2010) and suggests the need for scalability-focused formative research to be nested in a study to measure to effects of the intervention. In our study, we considered key dimensions of the scalability assessment to design the intervention theory of change—thus identifying potential barriers to feasibility and acceptability, and we plan to use the scalability assessment during pilot evaluation and at multiple points during the study, to refine our understanding of the optimal fit between intervention, implementation team, adopting organizations and socio-political context.

Thirdly, there was a tension between demonstrating proof of principle through a randomized controlled trial, and adapting the intervention to maximize its fit with the health system so as to aid scale-up, if proven effective. Waiting for the results of a multi-year trial before considering scale-up strategies, on the ground that proof of principle must be established first, is not a departure from common practice and leaves the scalability question unaddressed. Complex interventions are context-specific and therefore researchers and practitioners must consider attributes of the intervention, available capacities and resources required to produce impact at scale, once controlled study conditions end and adapt implementation over time. This may fit better with evaluation designs that allow for potential modification of the intervention during implementation, and may be hard to reconcile with randomized controlled trials, which often require fixed implementation protocols over multiple years, and monitor fidelity (or adherence to implementation protocols) to explain observed effects.

## Discussion

The limited literature on scalability suggests integrating scalability assessments into pilot projects. However, implementation does not always proceed linearly from pilot to scale-up (Craig *et al.*, 2008). Implementers are required to use ‘adaptive management’ approaches, that is to refine interventions to improve relevance and effectiveness as they are being implemented, while concurrently expanding coverage. In some settings, political pressure is such that small scale pilots are not encouraged (Spicer *et al.*, 2014). Evaluation is increasingly required in real time, and there are often pressures to scale-up promising interventions without conducting pragmatic trials or waiting for results of the pilot project evaluation (Indig *et al.*, 2017). For complex interventions, the distinction between proof of principle trial and implementation research is also more blurred. For example in our study, while each intervention component is underpinned by evidence derived from proof of principle RCTs (Chen *et al.*, 2018), it is also true that proof of principle is needed on whether the multi-component intervention would have the expected effects, and that it can be feasibly implemented (with opportunities for scale-up) in a LMIC setting.

The challenges presented above are not unique to QUALIDEC, and resonate with evaluation literature that has contrasted intervention-centric with context-centric approaches. There is a recognized methodological gap in methods and approaches to understand contexts in relation to effectiveness, and this also has implications for scalability, which can ultimately be thought of as an effort to maximize contextual fit (Craig *et al.*, 2008; Davey *et al.*, 2017).

Scale-up is a relatively new concept, often still conflated with replication and expansion. The body of literature on scale-up in implementation research is growing, but we found little evidence that the methodological challenges we have documented here have been fully addressed. Of the four scalability assessment tools we reviewed, two emerged from communities of practice (Cooley and Kohl, 2006; ExpandNet WHO, 2011), and experiences of moving from projects to programmes using the ExpandNet scalability assessment tool are increasingly being documented (Ghiron *et al.*, 2014; Keyonzo *et al.*, 2015; Omimo *et al.*, 2018). Implementation research has also documented intervention adaptation to aid scale-up of quality improvement interventions using the Institute for Healthcare Improvement's approach (Twum-Danso *et al.*, 2014; Barker *et al.*, 2016). These demonstrate the feasibility of using a scalability tool and framework to aid adaptive management, but do not provide evidence on whether an intervention that is gradually adapted to a context to aid scalability is more or less effective. In the research sphere, we found few studies that used the scalability tools identified in the peer-reviewed literature (Cambon *et al.*, 2013; Milat *et al.*, 2016) to consider the question of scalability of an intervention. Such studies were either retrospective case studies using the tool as an analytical framework (Trompette *et al.*, 2014; Vidgen *et al.*, 2018), or trial protocols proposing a qualitative implementation study or process evaluation focused on scale-up, running in parallel or at the end of the study (Kabore *et al.*, 2016; Lonsdale *et al.*, 2016). However, these are yet to generate evidence on the success of scaling-up strategies, as advocated by previous reviews (Ben Charif *et al.*, 2017).

Assessing and enhancing scalability compels researchers to engage with the concept of scalability from the start and undertake substantial formative research at baseline to design implementation protocols that maximize the potential for implementation at scale by considering the key scalability dimensions (attributes of the intervention design, the adopting community, the implementers and a fit with the socio-political context). It compels researchers to go beyond a one-off assessment during a pilot project (assuming there is one) (Cooley and Kohl, 2006; ExpandNet WHO, 2010; Barker *et al.*, 2016), and instead thoroughly document how the intervention or the way it was delivered evolved to enhance its scalability, for example through theory-driven and scale-up focused implementation studies running alongside a trial (Lund *et al.*, 2012). That is, to use more context-driven intervention and evaluation designs, with greater integration of impact and process evaluation, for which methods are advancing (Davey *et al.*, 2017).

An explicit focus on scalability also compels researchers to develop outcome analysis plans that take into account this evolution and compare interventions effects across phases of implementation, looking in these subgroups for evidence of whether the effects changed according to the phase, if adequate power can be reached.

We are fairly confident that the dimensions explored by our scalability assessment tool are comprehensive, because they incorporated all facilitating factors for scale-up emerging from our rapid review of scale-up frameworks. To our knowledge, none of the existing scalability tools have been validated, and content validity testing is beyond the scope of our study. However, we anticipate further refinement, including abbreviation of our tool as we begin using it, and later research may also test the tool’s predictive value.

## Conclusion

Achieving impact at scale is essential for the achievement of Sustainable Development Goals. The successful delivery of complex health interventions at scale requires a close fit between interventions, the socio-political contexts and the health systems in which they are implemented, which can be aided by early scalability assessments and ongoing scalability-focused implementation research. In this methodological musing, we described the process of incorporating scalability considerations in the design of study to evaluate an intervention to reduce unnecessary caesarean sections in low- and middle-income countries. We identified three key methodological challenges: achieving consensus on the purpose; identifying optimal timing; and resolving tensions between the need to establish proof of principle and the need to design an innovation that is fit-for-scale.

Partnerships between researchers and stakeholders are necessary to achieve sound contextual framing of a new intervention and to aid scale-up. The quality of these partnerships will determine both the extent to which health systems bottlenecks that may hinder scale-up can be debated in an open way during scalability assessments, and the extent to which interventions can be adapted to suit contexts.

We could not find evidence of studies that have fully resolved the methodological challenges we have documented; however, recently published study protocols are increasingly explicit about scalability considerations. We call for researchers to better incorporate scalability considerations in pragmatic trials through greater integration of impact and process evaluation, more stringent definition and measurement of scale-up objectives, and outcome evaluation plans that allow for comparison of effects at different stages of scale-up.

## Ethical approval

No ethical approval was required for this study.

## Supplementary Material

czz068_Supplementary_Annex

## Footnotes

1 The QUALI-DEC study is still under development and not yet registered.
