# Supplementary material for: Assessing scalability of an intervention: why, how and who?
Source: Health Policy Plan. 2019 Jul 31;34(7):544–52. doi: 10.1093/heapol/czz068 (PMC6788216; doi:10.1093/heapol/czz068)
Supplement: czz068_Supplementary_Annex [file czz068_supplementary_annex.docx]

Annex 1 – Definition of related concepts (1).

**Transferability** - the extent to which the effects of an intervention in a given setting can be observed in another population or context

**Replicability** - to the feasibility of implementation and acceptability of an intervention shown to be effective elsewhere in a new context, or with a new target group.

**Sustainability** – the extent to which an intervention can be continued beyond its initial implementation

1. Bonell C. Transfer and scale-up of health promotion interventions. In: Macdowall W, Bonell, C., and Davies, M., editor. Health Promotion Practice. Maidenhead: Open University Press; 2006. p. 220 - 30.
